# The shaping of a molecular linguist: How a career studying DNA energetics revealed the language of molecular communication

**DOI:** 10.1016/j.jbc.2021.100522

**Published:** 2021-04-07

**Authors:** Kenneth J. Breslauer

**Affiliations:** Department of Chemistry and Chemical Biology, Rutgers University, Piscataway, New Jersey, USA; The Rutgers Cancer Institute of New Jersey, New Brunswick, New Jersey, USA

**Keywords:** DNA energetics, energy databases, calorimetry, DNA, metastability, energy-based design, energy recognition/profiling, energy genome, rough DNA energy landscapes, energy: bridging structure and function, genetic code evolution

## Abstract

My personal and professional journeys have been far from predictable based on my early childhood. Owing to a range of serendipitous influences, I miraculously transitioned from a rebellious, apathetic teenage street urchin who did poorly in school to a highly motivated, disciplined, and ambitious academic honors student. I was the proverbial “late bloomer.” Ultimately, I earned my PhD in biophysical chemistry at Yale, followed by a postdoc fellowship at Berkeley. These two meccas of thermodynamics, coupled with my deep fascination with biology, instilled in me a passion to pursue an academic career focused on mapping the energy landscapes of biological systems. I viewed differential energetics as the language of molecular communication that would dictate and control biological structures, as well as modulate the modes of action associated with biological functions. I wanted to be a “molecular linguist.” For the next 50 years, my group and I used a combination of spectroscopic and calorimetric techniques to characterize the energy profiles of the polymorphic conformational space of DNA molecules, their differential ligand-binding properties, and the energy landscapes associated with mutagenic DNA damage recognition, repair, and replication. As elaborated below, the resultant energy databases have enabled the development of quantitative molecular biology through the rational design of primers, probes, and arrays for diagnostic, therapeutic, and molecular-profiling protocols, which collectively have contributed to a myriad of biomedical assays. Such profiling is further justified by yielding unique energy-based insights that complement and expand elegant, structure-based understandings of biological processes.

I was born in Jönköping, Sweden, the third child of parents who were refugees from Nazi Germany and who eventually settled in New York City. My mother was a Columbia University–trained social worker who always provided me with gentle nurturing and encouragement. My father was a frustrated academician who became a businessman in New York. In stark contrast with my mother, my father was strict and demanding. Much to my father's delight, my older sister and brother both were academic superstars. By contrast, much to my father's consternation, I was a “street urchin” who was far more interested in sports than academics.

I was “saved” by my high school baseball coach, Dr Edson Scudder, a Princeton PhD, who taught history. He told me that unless I improved my grades, he would not allow me on the baseball team. Each day, Dr Scudder would stay after school and tutor me. My grades soared, as I became the only person in the high school to go in one semester from academic probation to the academic honor roll! For the rest of my high school years, I started in left field and won all-conference honors. Despite my relatively weak arm, I was a very good hitter. I went on to college and majored in history because of Dr Scudder's influence. I also majored in chemistry because I wanted eventually to become employed. I believe I was the only such double major that year at the university.

From these somewhat uneven beginnings, I never could have envisioned a day when I would write a *Reflections* piece of my nearly 5 decades of DNA scientific research. My personal story proves that, in the race of life, not all winners take the lead immediately out of the starting gate. I am convinced this is why I never give up on any of my students, knowing that they also may prove to be late bloomers. I am humbled and honored to be able to share with you my personal and professional reflections.

As a teenager growing up in New York City, I had three passions: baseball, history, and science. Brief stints playing college and minor league baseball convinced me to focus my attention on the other two passions. As noted above, I earned a BA in history and a B.S. in chemistry from the University of Wisconsin at Madison, graduating in 1968. The Vietnam War and the associated draft sharply shaped the next prioritization of my career. Science would become my vocation, and history would be my avocation, as they both remain so to this day.

In graduate school at Yale, I began as a physical organic chemist. Under the mentorship of Professor Jerome A. Berson, I investigated terpene rearrangements. The goal was to test the limits of the orbital symmetry properties that formed the underpinnings of the Nobel Prize–winning Woodward–Hoffmann rules. At the same time, I took an elective course in physical biochemistry, taught by Joe Fruton and Fred Richards. This course had a profound impact on my scientific trajectory. I was fascinated to learn how the three-dimensional shapes and functions of large, complex biological compounds, including polymers, could be understood in terms of fundamental thermodynamic and kinetic properties. The lecturers frequently underscored that all biochemical events are controlled and regulated by differential energetics, which are manifest in maps of the isolated and interacting energy landscapes. In essence, favorable and unfavorable interaction energies constitute the words of the molecular language by which molecules communicate and orchestrate their structures, stabilities, and functions, or dysfunctions. I was smitten!

I went on to pursue my PhD studies in biothermodynamics and kinetics at Yale University with Julian Sturtevant (1), followed by a postdoc at the University of California, Berkeley, with Ignacio “Nacho” Tinoco Jr. (2), and finally, in 1974, a faculty position at Rutgers University, where I remain to this day.

During my first 5 or 6 years at Rutgers, I split my time between intramural softball with the students and setting up my laboratory, where I conducted research in pursuit of nature's secrets of life, as well as in pursuit of tenure! I have been at Rutgers for 47 years, during which I became a husband, a father, the Linus C. Pauling distinguished university professor, the founding dean of the Division of Life Sciences, and vice president for health science partnerships. Currently enabled by an outstanding group of colleagues in my laboratory, I continue my biophysical research programs, which collectively focus on characterizing the forces that modulate the regulation and dysregulation of biological processes crucial to human health.

My laboratory at Rutgers has spent nearly 5 decades creating DNA databases that systematically characterize the intramolecular and intermolecular forces, which collectively constitute the words, sentences, paragraphs, and chapters in the book of molecular communication. As such, one could reasonably characterize me as a molecular linguist.

This scientific focus was shaped within me by my early training at Yale from 1968 to 1972. Julian Sturtevant (1) and Donald Crothers (3) unrelentingly emphasized to me, and others, the importance of quantitatively characterizing the energy profiles of molecular assemblies to understand the origins of the structures, as well as the modes of action of their functions. I was surprised by how quickly I became a convert to their mantras because as a student I observed that no single word in the scientific lexicon could more rapidly empty a classroom than “thermodynamics.” However, conversations and classes with them, as well as with Lars Onsager, Oktay Sinanoğlu, Kenneth Wiberg, and others, made energetic profiling my passion rather than my fear. My conversion was further facilitated by Don Crothers asking me to proofread and propose edits for a textbook manuscript that he, Ignacio Tinoco, and Victor Bloomfield were writing (4). I was mesmerized by the quantitative rigor and clarity of explanations presented in the text, and I felt privileged to have such a sneak peek before publication. This opportunity was coupled with the 1973 publication of the Tinoco/Crothers landmark paper “Improved Estimation of Secondary Structure in Ribonucleic Acids” (5), and I became even more convinced of the need for further energy studies on biological systems. In 1973, Douglas Turner and I were postdocs in the Tinoco laboratory, during which time we became close friends; although I still cannot get over the fact that he bested me 1-0 in a game of stickball! It is interesting to note that when we accepted independent faculty appointments, Doug built his stellar academic career on an impressive and much-needed elaboration of RNA energy profiling (referred to as the “Turner Numbers”) (6), while my academic career has focused on establishing DNA energy databases, including their drug- and protein-binding properties.

My choice of research programs as well as my deep commitment to data collection were profoundly influenced by my years in Sturtevant's laboratory at Yale (1). I fondly recall a conversation with Julian in which I showed him my voluminous tabulation of calorimetric data on a biological system and lamented that I could not discern any pattern that would glean new insights. He gently smiled and said, “That only means you haven't yet collected enough data, since the patterns will become self-evident when you reach the critical data density.” From that moment forward, I committed myself to a career of databasing, of ever-increasing density, on biologically relevant molecules. It therefore should come as no surprise that as a dean and vice president, I recruited Helen Berman, who established at Rutgers the international structural database (Research Collaboratory for Structural Bioinformatics Protein Data Bank, or RCSB EDB) (7, 8) and subsequently recruited her successor, Stephen Burley, as the director (9). During my early years, in the mid-1970s to early 1980s, I also was influenced by the published works of and conversations with Walter Kauzmann, Rufus Lumry, Peter Privalov, Harold Scheraga, Charles Tanford, and other luminaries. These interactions further convinced me of the importance of an energy database focus for my own research. While Tinoco, Crothers, Turner, and others focused on RNA energetics, I decided that direct, model-independent, calorimetric characterizations of DNA energetics was needed. My postdoc project, published in the Journal of Molecular Biology in 1975 (10), introduced the Tinoco group to the added insights derived from a differential scanning calorimetric mapping of the energetics of an oligomeric ribo duplex. Based on this demonstration, coupled with improvements in DNA synthesis and commercially available, high-sensitivity calorimeters, Tinoco, Crothers, and Sturtevant encouraged me initially to focus my own laboratory work on DNA energetics, as elaborated below.

During my first decade at Rutgers (1974–1984), I pursued a range of calorimetric studies on specially designed and synthesized DNA oligomers of systematically varying sequences. From these measurements, and inspired by the work of Tinoco and Crothers (5, 11, 12), I was able to derive the first direct, model-independent, calorimetrically derived DNA thermodynamic library of sequence-dependent, nearest-neighbor interactions (13)—a library that quickly became used throughout molecular biology to rationally design DNA primers and probes. During my second decade and a half at Rutgers (1984–2000), my laboratory increased the data density of this DNA library to include many noncanonical, biologically relevant altered features of DNA forms, such as triplexes, tetraplexes, bent DNA, crosslinked DNA structures, ligand-perturbed DNA structures, and drug-bound structures. Overlapping this period up to the present, we have focused on mapping the rough energy landscapes of metastable DNA states, as well as characterizing lesion-induced alterations in DNA properties and how these perturbations correlate with biological recognition, processing, repair, mutagenesis, cytotoxicity, and other biologically relevant properties.

In my filing cabinet at Rutgers is an artifact reflecting my laboratory's growing recognition in biothermodynamics. The folder is titled Kauzmann v. Lumry.

They were very close friends; in fact, Rufus was Walter's best man at his wedding. However, their friendship was tested in the late 1970s, as they exchanged heated arguments about thermodynamics. They finally asked me to serve as an intermediary for their correspondence to avoid making their letters too abrasive. I was both honored and intimidated. I refrain from sharing further substance of this file, given my deep respect for both individuals and my confidential role as an impartial arbitrator! I will say, however, the experience validated me in the eyes of two giants in the field, further cementing my confidence to pursue a career dedicated to mapping the energetics of biological systems.

In this reminiscing *Reflections* article, I provide select examples of how, starting in 1974, my laboratory at Rutgers, and, previously and/or contemporaneously, the laboratories of others (*e.g.*, Ignacio Tinoco, Don Crothers, Victor Bloomfield, Peter von Hippel, David Mathews, Doug Turner) were demonstrating that nucleic acid energy profiles, both thermodynamic and kinetic, provide the missing links between DNA and/or RNA biological structures, differential stabilities, and biological functions.

## Energy profiling: The missing link

The experimental and computational mapping of energy profiles that bridge the conceptual and practical gaps between biopolymer structures and biological functions is motivating a generation of young scientists to pursue bioenergetic studies. As elaborated in this article, such profiling is justified by yielding unique energy-based insights that complement and expand elegant, structure-based understandings of biological processes.

Enhanced reporting of energy profiling, both thermodynamic and kinetic, reflects the expanding recognition that such fundamental data are essential for foundational understandings of molecular recognition, as well as for the establishment of new biomedically relevant applications. In fact, DNA energy databases have enabled a revolution in quantitative molecular biology through the rational design of primers, probes, and arrays for diagnostic, therapeutic, and molecular-profiling protocols that have contributed to a myriad of biomedical assays. The future is bright for those physical, computational, and structural chemists who also choose to focus on characterizing the energy landscapes of complex biological assemblies.

As noted above, energetics provides the bridge between biological structure and biochemical function. International genomics and proteomics initiatives, empowered by technological and methodological advances, have produced a wealth of crucial structural and functional data (7, 9, 14). In contrast, the mapping of the energy landscapes that link these structures with their biological functions has lagged. This deficiency is manifest in and inhibitory of numerous biomedical research efforts, including, but not limited to, energy-based drug design (EBDD), hybridization-based gene regulation, mechanisms of DNA repair, differential protein stabilities and their folding/misfolding pathways, rational protein engineering, and many other examples. This article is not intended to discuss all such areas but rather to select several DNA systems that exemplify the value of energetics as the essential link between biological structure and function. To this end, this article primarily will use DNA examples from my own laboratory, while acknowledging that many other laboratories have made important contributions to building such energy bridges, both computationally and experimentally, for other biological systems.

## The impact of collaborative science without borders

Over the years, my laboratory's studies have been greatly influenced and enriched by interactions with visiting luminaries, as part of distinguished lecture series, as well as by national and international collaborations with visiting scientists on sabbatical, followed by the hiring of some of their best students. To be specific, in the late 1960s to early 1970s, the Peruvian government sent some of their best young scientists to the United States for training. I was fortunate enough to have one, Dr Luis Marky, join my group and take the lead on several projects, including calorimetric determination of our original, nearest-neighbor DNA database compilation. True to the collaborative nature of my group, we were able to achieve the requisite data density and diversity for these studies by studying specially designed oligomeric and polymeric DNA samples produced and provided by a German research team led by Helmut Blöcker and Ronald Frank. Clearly, it took an international scientific village to produce the first calorimetrically determined, nearest-neighbor DNA database (13).

In this same collaborative spirit of science without borders, we pursued additional energy profiling, including drug–DNA mapping, empowered by cooperative, sabbatical-based joint ventures with Slovenian scientists, Professors Gorazd Vesnaver and Ciril Pohar, as well as Natasa Poklar, all from the University of Ljubljana; with Czech Republic scientists, Professors Bohdan Schneider and Ctirad Hofr; with German and South African scientists, Professors Horst Klump and Jens Völker; with Armenian and Russian scientists (from the former Union of Soviet Socialist Republics), Professors Armen Sarvazyan, Tigran Chalikian, and Vera Gindikin; with Chinese scientists, Dr Renzhe Jin (a Tiananmen Square refugee) and Wan-Yin Chou; with a South Korean scientist, Dr Young-Whan Park; with Polish scientists, Dr Danuta Szwajkajzer and Slawomir S. Mielewczyk; with a Brazilian scientist, Professor Cica Minetti; as well as with United States–based scientists, including Drs. Eric Plum, Craig Gelfand, William Braunlin, and Scott Law and Professors David Remeta, Dorothy Erie, Arthur Grollman, Francis Johnson, Dani Pilch, Ron Breslow, Peter Dervan, Roger Jones, Barbara Gaffney, Gary Glick, Nick Geacintov, Doug Turner, Jerry Manning, Wilma Olson, and many others.

One of the goals of this article is to illustrate selectively the need for fundamental thermodynamic measurements on complex DNA systems, while also acknowledging the significance of kinetic factors that further shape energy landscapes; particularly, as manifest in suboptimal, metastable states that can serve as regulatory switches, a feature reflected in the work of Jens Völker, who is now a research professor in my group at Rutgers. In short, DNA energy profiling enables the establishment of essential linkages between high-profile DNA structures and crucial biological functions/pathways. By underscoring the enhanced insights derived from such DNA energy mapping, a generation of classically trained physical chemists may be motivated to apply their invaluable expertise to clarify the complexities of biological systems in terms of fundamental physiochemical principles. In so doing, they would greatly enrich the scientific arsenal of the biophysical community.

## Calorimetry: The experimental method of choice

My introduction to calorimetric instrumentation was less than encouraging; in fact, it bordered on the traumatic, as I explained in a published tribute to the memory of Julian Sturtevant (1). It was late 1968/early 1969, and I was beginning my PhD studies at Yale in the Sturtevant laboratory. One evening, during my first few months there, I detected some erratic behavior by the flow calorimeter. As I wrote in the tribute to Julian (1):

It appeared that some moisture had breached the sealed submarine in which the heat sink and flow tubing were embedded. With some trepidation, I called Julian at his home, anticipating that he would tell me to stop and that he would look at it in the morning. Instead, Julian directed me to dissemble the instrument, place the heat sink in a drying oven, and then reassemble the unit in the morning. I politely listened, but I was terrorized by this directive, having never even seen the immersed calorimeter beyond the motor-driven syringes I used to deliver the reactant solutions. When I shared my fear with Dr Sturtevant, he chuckled and said ‘You should know what the insides of the instrument looks like and how it works. Only then will you understand what you really are measuring so you can interpret your data and design new experiments in an informed manner.’ I proceeded to dissemble the instrument, with the screwdriver shaking in my hand. I finally got to the heat sink, disconnected the flow tubing, and I placed the aluminum block in the drying oven. I then returned to my desk to enter all this information into my laboratory notebook. An hour later, I opened the drying oven and looked inside the heat sink. To my amazement and terror, I saw a pool of a reflective, silvery liquid that looked like melted metal. Had I just destroyed the instrument by melting the flow tubing? My terror prevented me from realizing that the temperature of the gentle drying oven was very far below the melting point of platinum or any other material used for construction of the instrument. I decided that I had to call and tell Dr Sturtevant what transpired, despite fearfully envisioning next day headlines proclaiming the shortest tenure of a graduate student in the history of Yale University. When I apologetically confessed to Dr Sturtevant what had happened, he roared with laughter and told me that the melted material was just ‘woods metal’ used to enhance thermal contact and that everything was fine. I instantly became a Julian Sturtevant fan for life.

Given this maiden voyage with calorimetric instrumentation, it is a true testament to Julian's sensitivity and humanity that I have made this class of instruments the cornerstone of my research career.

It has been long recognized that calorimetry represents the most direct, model- and label-free experimental approach for obtaining the energy data of interest. However, for decades, most commercially available calorimeters lacked the sensitivity required for detecting the small amount of heat accompanying biological studies. A few laboratories, most notably those of Julian Sturtevant, Peter Privalov, Rufus Lumry, and later John Brandts and Rod Biltonen built one-off, more sensitive microcalorimeters to conduct their biothermodynamic measurements, yet these instruments initially were not commercially available, and they lacked the throughput desired for many applications. Motivated by these limitations, specialty companies such as MicroCal, Calorimetry Sciences Corp., Tronac, Malvern Panalytical, and Thermal Analysis began to commercialize highly sensitive microcalorimeters, thereby enabling an increasing number of laboratories capable of performing bio-level measurements. The next technical hurdle was to increase throughput, a challenge that has been surmounted *via* automation and multicell configurations, thereby making the methodology far more appealing to drug discovery/material science screening efforts.

Armed with this new generation of microcalorimeters, one now has the ability to map the subtleties of energy landscapes, including metastable states and conformational switches that are at the foundation of regulatory events in biology and thus are essential for the rational design of physiochemical properties in material sciences. Physical chemists know that energetics represent the universal language of molecular recognition. Likewise, biophysical chemists know that energetics are required to bridge the conceptual and practical gaps between structure and function. Through the ongoing efforts of both overlapping communities, energy databases are beginning to become sufficiently populated to allow for fundamental and predictive correlations to be established between biophysical properties and biological function. What follows is a sampling of examples that illustrate the need for and enhanced insights produced by energy profiling of DNA biological systems.

## DNA thermodynamic databases: Rational design of primers and probes

PCR is a method that allows one to make billions of copies of originally minute amounts of target DNA samples of interest (15). This amplifying technology revolutionized a myriad of DNA-based applications, including molecular diagnostics and therapeutics, forensic molecular fingerprinting, DNA cloning, and mapping of DNA-based phylogenies, while enabling multiple other fundamentally important areas of inquiry. A key reagent in PCR is known as a primer. Primers are relatively short pieces of single-stranded DNA that are Watson–Crick complementary to a target region(s) on one strand of the DNA to be amplified. The resultant hybridized primer-DNA target strand mini-duplex, with the overhanging single strand, then serves as a template for a DNA polymerase enzyme to produce a DNA copy. As this chain reaction cycles, it yields billions of copies of the original minute DNA sample. To optimize the error-free fidelity of this amplification technique requires a rational basis for the primer design that goes beyond simple Watson–Crick hybridization and which includes defining PCR conditions that maximize stringency. To this end, the need for sequence and solution-dependent thermodynamic data became self-evident. The requisite nearest-neighbor thermodynamic databases for Watson–Crick domains (13, 16, 17, 18, 19, 20, 21, 22, 23) were determined by multiple laboratories, including those of Tinoco (5, 11), Crothers (12), Turner and Mathews (6, 24, 25), as well as my own, and they continue to be improved upon. The resultant data, including more recently reported heat capacity effects (26, 27, 28, 29, 30, 31), have been incorporated into commercial and proprietary algorithms used worldwide to enable differential stability-based design of hybridization-based diagnostic and therapeutic protocols (32, 33, 34, 35, 36).

These energy databases also characterize DNA structures that contain biologically consequential mismatches (37, 38), modified bases/lesions (37, 39, 40, 41, 42, 43, 44, 45, 46, 47), as well as nonduplex secondary structures, including the energetics of triplexes (48, 49, 50, 51, 52), tetraplexes (53, 54, 55, 56) (associated with modulating telomere stability and epigenetic modifications) (57, 58), and higher order, functionally relevant DNA assemblies (59, 60, 61, 62, 63, 64). My laboratory's triplex DNA studies were performed in collaboration with Peter Dervan's group, whom I knew well from our time together as graduate students at Yale. He sent one of his students, Scott Singleton, to my laboratory at Rutgers to learn calorimetric techniques for measuring the energetics of triplex formation, an assessment he needed to tune the stringency of his designed, groove-binding synthetic endonucleases (49). My laboratory's subsequent focus on mapping the energetics of tetraplex complexes (53, 54) was stimulated by Alex Rich meeting with me and my group as part of his seminar visit to Rutgers. By the end of the day, he had convinced most members of my group to drop their projects and pursue energy mapping of tetraplexes and Z-DNA! Likewise, a seminar visit from Peter Schultz resulted in a postdoc offer for a graduate student in my laboratory, accompanied by lobbying me to allow my student to complete his thesis expeditiously so he could get to Berkeley as soon as possible. In 1991, the Merck lecture at Rutgers was given by Ron Breslow of Columbia University. In a separate meeting with me and my group during his visit, Ron asked if any of us could explain why DNA “evolved” to use 5′-3′ rather than 5′-2′ phosphodiester linkages. Without waiting for our speculation, he proclaimed that he had developed a synthetic pathway for the 5′-2′ links and wanted us to conduct a comparative thermodynamic profile of it with the base sequence homologous 5′-3′ counterpart. I agreed that it would be a very interesting comparison. Ron bellowed, “Do you think the world is ready for a Breslow–Breslauer Proceedings of the National Academy of Sciences paper?” I responded, “Probably not, but I am confident that they are ready for a Breslauer–Breslow paper.” He roared with laughter, we performed the work, and we published the fascinating results in Proceedings of the National Academy of Sciences in 1993 (65). We scored another victory for collaborative science.

To date, the most important applications of the expanding DNA databases include the following: the differential stability–based rational design of nucleic acid primers and probes with enhanced stringency that minimizes false positives and false negatives, in both clinical and forensic assays (66, 67); the rational modification and conversion of oligonucleotides into sequence-specific antisense and antigene agents, including synthetic endonucleases (68, 69, 70, 71, 72, 73, 74, 75); and the rational design/configuration of DNA oligonucleotide arrays on high-throughput chips for molecular profiling (76, 77). In sum, these DNA energy databases have helped revolutionize quantitative molecular biology through the energy-based design of primers, probes, and arrays for diagnostic, therapeutic, and molecular-profiling protocols that have contributed to the rational design of a myriad of biomedical assays.

## “Energy recognition”: isostructural does not necessarily mean isoenergetic

**Figure 1:**
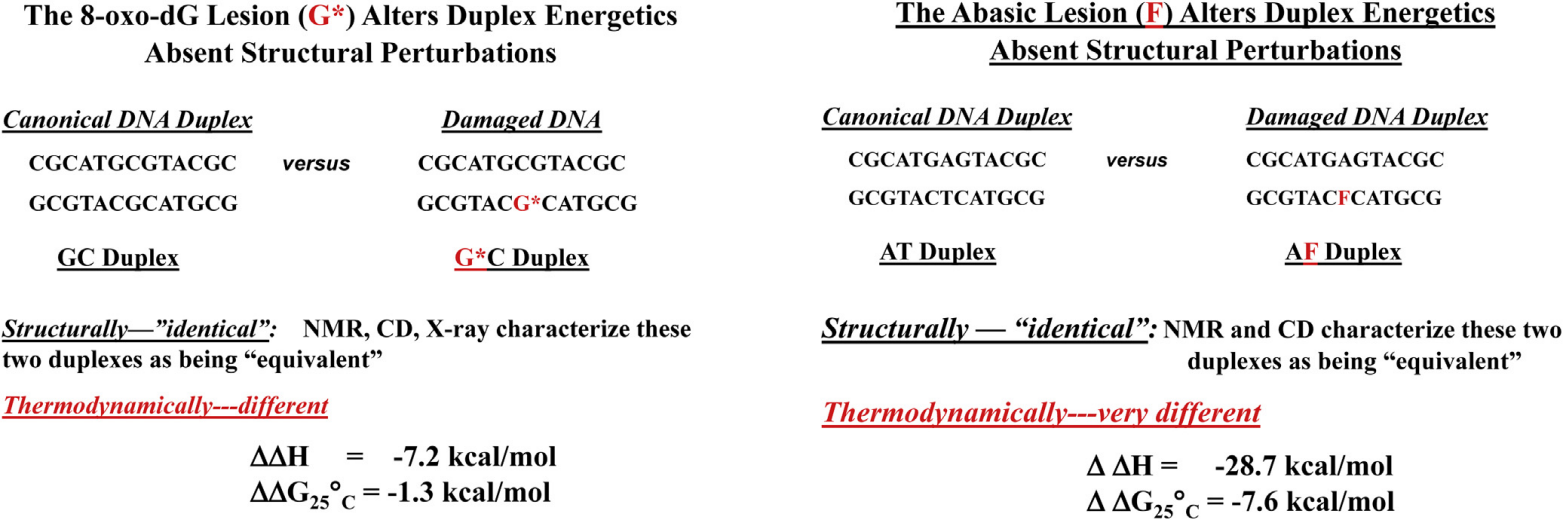
**Examples of isostructural does not necessarily mean isoenergetic.**

Throughout the 1980s and 1990s, my laboratory was part of a National Cancer Institute–funded program with Arthur Grollman's group at Stony Brook University, on DNA recognition, repair, and mutagenesis. A central component of our project focused on comparing the properties of oligomeric DNA duplexes both with and without lesion sites. A consistent observation from our studies revealed that DNA lesions can have significant consequences on DNA energetics, even in the absence of detectable levels of lesion-induced structural/conformational alterations. In other words, *isostructural does not necessarily imply isoenergetic*. Two examples of this phenomenon are illustrated by the data in Figure 1 (42, 44). Note that the canonical and corresponding lesion-containing duplexes (with either an 8-oxo-dG or abasic “damaged” site) exhibit indistinguishable duplex forms, insofar as the global conformations as defined by CD, NMR, and/or X-ray measurements are concerned, yet they manifest substantial differences in their energy profiles.

The examples in Figure 1 underscore the need for comparative structural studies in parallel to the corresponding energy characterizations. Furthermore, the magnitudes of these structurally/conformationally “silent,” lesion-induced energy perturbations were consistent with their destabilizing impact beyond the lesion site to create domains of genome instability (37, 39, 40, 42, 78). We proposed that such energetically perturbed domains may serve as initial DNA recognition elements for the machinery of repair, a phenomenon we referred to as *energetic recognition* (79). Such initial sensing of an energy-perturbed domain, as the processing repair enzyme surveils DNA for damage, is envisioned to occur before the more localized, site-specific protein interactions with distinct structural features of the lesion. Consequently, mapping energy landscapes of lesion-containing DNA substrates, and their protein-binding properties, is essential for understanding lesion recognition, repair, and translesion DNA synthesis—including the failure of these pathways that leads to mutagenesis. Knowledge of energy profiles/mechanistic pathways of repair (*e.g.*, base excision repair, or BER) is required for the rational design of intervention strategies that target specific steps/species/checkpoints encountered in such processing pathways, such as abasic sites, damaged/modified bases, altered secondary structures, bulges, and mismatches.

## Repair enzyme differential binding affinities: Preferential binding for product over substrate

**Figure 2:**
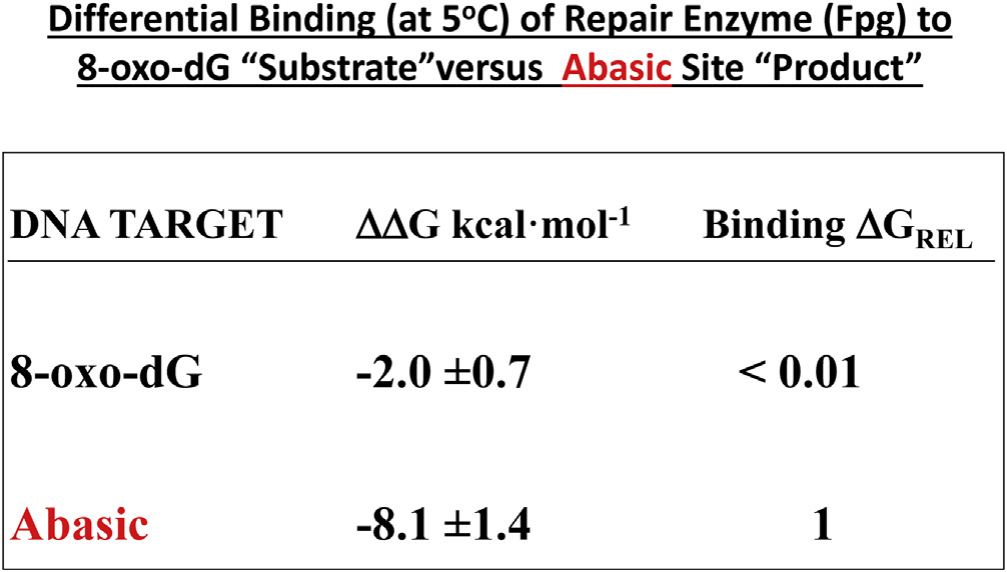
**Example of preferential binding of a processing enzyme for the product over substrate.**

A logical next step in our contribution to the program was to probe differential repair enzyme binding to duplexes with the lesion and with the lesion excised by a DNA glycosylase. Before our promulgation of the concept of energetic recognition, it was broadly assumed that an enzyme would exhibit the greatest affinity for its nascent substrate. However, our energy studies revealed, counter to this expectation, that an endonuclease can bind more strongly to the highly destabilizing abasic site product generated by lesion processing, in contrast with its relatively weak binding to the only marginally destabilizing 8-oxo guanine nascent substrate target (80, 81, 82). Figure 2 illustrates the relevant data for one system (79).

This unexpected differential affinity required energy mapping to discern and would not have been evident from structural studies alone. Such an energy-based insight supports a novel hypothesis that enzymatic excision of a damaged base can be driven by a repair enzyme binding more strongly to the product than to the substrate. One can view this differential affinity as another example of *energy recognition*, as the product-containing abasic site is far more destabilizing than the substrate-containing 8-oxo guanine site. This insight is consistent with the hypothesis that lesions can induce domains of altered stability that may serve as recognition sites for repair proteins (46).

In summary, under nonturnover conditions, a DNA glycosylase can bind more strongly to a highly destabilized duplex containing the base excision–derived abasic product analog than to the less-destabilized duplex containing the nascent lesion substrate analog (79, 81). This finding suggests that protein–DNA interactions during catalysis can favor transition state/product binding/stabilization over substrate binding/stabilization, thereby driving the catalytic event. The preferred binding to the abasic product/intermediate also serves to sequester mutagenic abasic sites before subsequent repair steps. Beyond providing insights into protein–DNA binding and catalysis, such *energetic recognition* has implications in the rational design of inhibitors and antagonists of DNA-processing pathways.

## Energetics as signatures of differential biological consequences

The connectivity between DNA thermodynamic studies and carcinogenesis is reflected in the energy profiles/signatures of DNA mutagenic lesions and their recognition and processing by the cellular repair machinery (80, 83, 84, 85, 86, 87). Secondarily, these studies also reinforce the concept of *energy recognition* in which DNA-repair enzymes sense regions of lesion-perturbed DNA stability before contacting structurally localized lesion sites (39, 42, 46). This concept is consistent with the hypothesis that nearly all lesions induce energy penalties that propagate several base pairs beyond the 5′ and 3′ sides of the lesion. For the processing repair enzyme, this propagated perturbation creates recognizable islands/domains of instability distal from the lesion site, before the more direct and structurally dictated encounter with the localized lesion site.

Here is an additional example of the enhanced insights derived from thermodynamic characterizations: it has been postulated that perturbed energetics of damaged duplexes serve as predictors of the biological consequences exhibited by the DNA lesion (*e.g.*, mutagenicity *versus* cytotoxicity) (88, 89). Specifically, the replication repair machinery engages template-primer substrates and may either bypass/misread a damaged base, such as the mutagen 8-oxo-dG (42, 79, 90), or stall at a potentially cytotoxic site, such as thymine glycol (40, 91). As the requisite energy data density and diversity increases, more biological outcomes should be predictable based on the energetic signatures of the DNA lesions embedded in their respective host duplexes. Again, in these measurements, parallel structural studies reveal that *isostructural does not always imply isoenergetic*, a fundamental insight that requires energy profiling to avoid missing DNA lesion–induced energy perturbations that may be structurally “silent.” This maxim has been demonstrated to be true in both nucleic acid and protein systems. Taken together, these results enable energy- and product-based design of exogenous agents that can modulate DNA repair, a major pathway that is dysregulated in cancer initiation and progression. Of course, it also is true that *isoenergetic does not necessarily imply isostructural*, an observation that may reflect significant enthalpy–entropy compensations that compress free energy distinctions. In such cases, one detects the energy differences in one or more of the other thermodynamic parameters.

There are many other examples of energetics as putative signatures of differential biological consequences. For instance, frameshift mutagenesis frequency correlates with the differential stabilities of bulge-looped duplex structures that serve as intermediates for insertion and deletion mutations (92, 93). Consequently, energy profiling of single-base bulged structures (37) is of direct biological relevance, while also providing an energy database for predicting potential hotspots in the genome. It has been postulated that the differential energy profiles of lesions as a function of the lesion type, sequence context, and counterbase correlate with repair success and failure (39, 43, 44, 94), thus providing a basis for rationalizing differential mutagenicity frequencies. Further energy profiling of lesion-containing DNA constructs is required to more robustly test this hypothesis.

## Energy profiling of DNA recognition, repair, and replication

DNA recognition, repair, and replication (the three R's of DNA) represent interrelated DNA processing events of central biomedical significance, particularly because errors in any of these three steps can result in mutational events that might trigger physiological disorders. In advocating for parallel energy and structural studies, I previously have emphasized that DNA lesions can significantly alter DNA energetics, even in the absence of detectable levels of lesion-induced structural/conformational perturbations. Consequently, mapping energy landscapes of lesion-containing DNA substrates, and their protein-binding properties, is essential for understanding lesion recognition, repair, and translesion synthesis—including the failure of these pathways, which can lead to mutagenesis.

**Figure 3:**
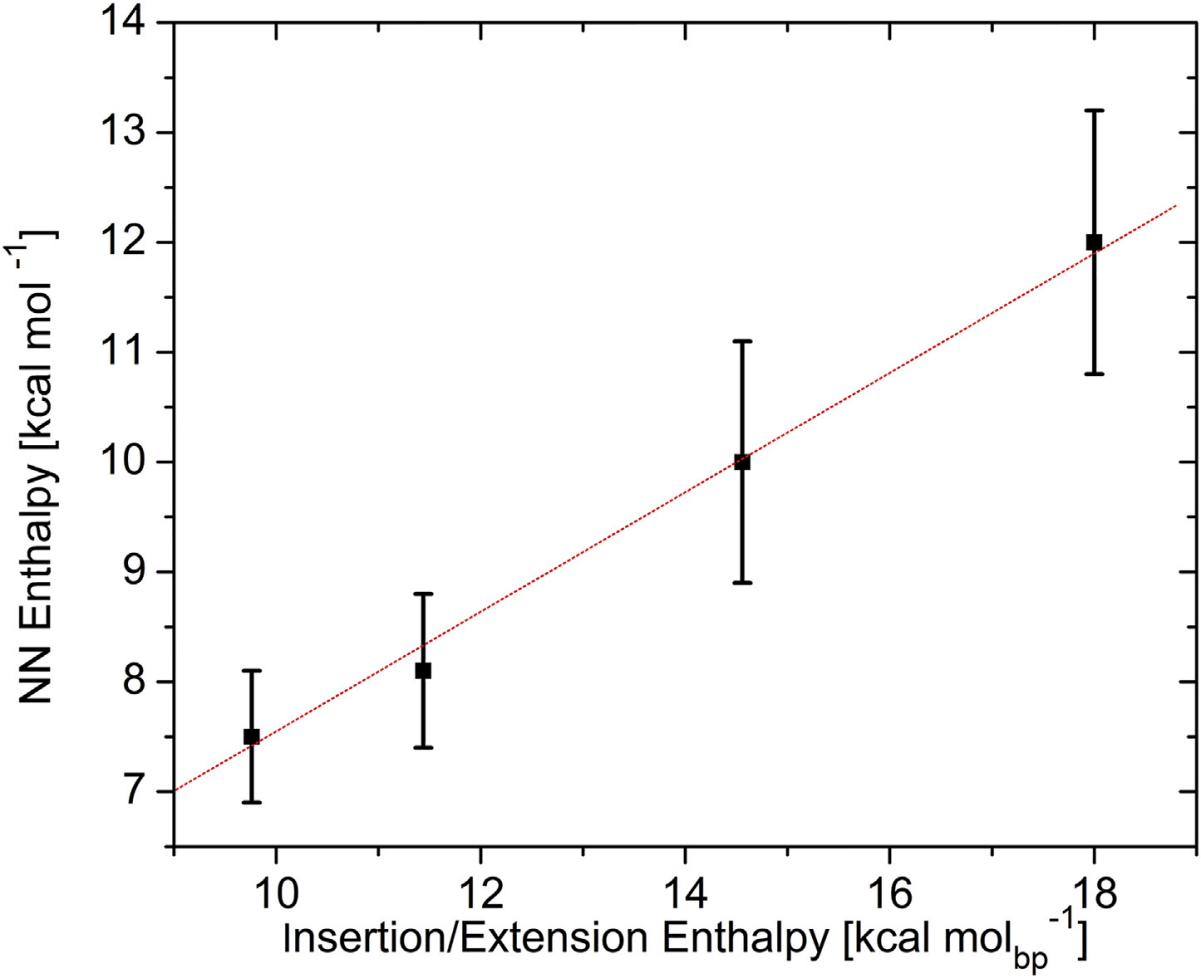
**Average nearest-neighbor enthalpies*****versus*****base insertion/extension enthalpies.**

In this context, it has been proposed, and in some cases demonstrated, that the differential stabilities of lesions as a function of their opposite bases dictate and control error-free *versus* error-prone translesion synthesis (95). As a result, energy profiling of lesion-induced destabilizations as a function of the opposite base relative to the corresponding undamaged pair/mispair (46, 96) is of direct biological relevance for predicting relative insertion frequencies, as well as predicting lesion-mediated mutagenicity *versus* adduct-induced genotoxicity. Knowledge of energy profiles/mechanistic pathways of repair (*e.g.*, BER) also is required to guide the design of intervention strategies to target specific steps/species/checkpoints encountered in such processing pathways. Intriguingly, under the leadership of Research Professors David Remeta, Cica Minetti, Eric Plum, and Craig Gelfand, we have demonstrated a correlation between polymerase-mediated insertion/extension enthalpies (80) and the corresponding nearest-neighbor values in DNA databases (13, 16, 17, 18, 19, 20, 21, 22, 23), as illustrated in Figure 3.

Thus, despite the making and breaking of covalent bonds during the replication process, due to large compensatory effects, only modest noncovalent nearest-neighbor forces control replication efficiency and fidelity in a predictable sequence-dependent manner. In short, base insertion discriminations are dictated by modest energy differences in noncovalent interactions at the template-primer level that correlate with the corresponding nearest-neighbor data. In addition to providing the framework for the design of sequencing platforms that employ thermoelectric high-throughput microfluidics initiatives (96), such data also set the stage for studies of sequence-dependent energetics for polymerase-template DNA interactions and catalysis, both during normal replication and during translesion synthesis. The resultant energy profiles allow predictions of the relative probabilities of specific base insertion as a function of the polymerase and the canonical base or lesion in the template.

## DNA-repair pathways, energetics, and cancer

There is an emerging literature on DNA-repair pathways as targets for cancer therapy (97, 98, 99), which underscores how mechanistic/energetic mapping of such pathways provide the basis for developing such therapy (86, 100) and cancer treatment protocols (101) [as reviewed (102)]. In fact, DNA-targeting drugs represent a large proportion of the available anticancer pharmacopeia (103). DNA-interacting molecules share the ability of certain DNA-binding proteins to perturb both DNA structure and energetics, some of which display helix-destabilizing properties. The formation of such locally destabilized DNA domains is likely to interfere with protein–DNA recognition, thereby potentially affecting DNA repair, DNA replication, and transcription (103). This property may be exploited in antitumor therapeutics. To enable such strategies, energetic profiles are required to map oxidative, exocyclic, and drug-induced DNA lesion recognition/repair pathways, as a prerequisite for defining the appropriate targets for modulating biological outcomes. This work in my laboratory, originally inspired by interactions with Professors Dinshaw Patel and Arthur Grollman, has been, and continues to be, led and conducted by Remeta and Minetti at Rutgers.

Existing thermodynamic data suggest that sequence-dependent energetic signatures of DNA lesions may serve as diagnostic probes of promutagenic *versus* cytotoxic propensities of specific adducts, the latter feature being a desirable property in some anticancer interventions. Furthermore, as a probe of miscoding and/or lesion-blocking properties, energy data help define the basis for mechanisms of chemical-induced carcinogenesis (104) and/or radiation-induced cell death (105). Other applications of energy data include delineating the origins for failure in anticancer therapeutic interventions (*e.g.*, chemical- or radiation-induced DNA adduct formation and consequent damage persistence) (106, 107), as well as mechanisms associated with metabolic imbalances and age-dependent neurodegenerative processes (108, 109).

Sequence context–dependent lesion energetics are also valuable for identifying and defining the origins of biologically consequential hotspots within the genome. Differential energy profiles may provide a metric for risk assessment of environmental exposures or metabolic anomalies. Calorimetric evaluations of the requisite energetics associated with template-directed polymerase-mediated synthesis at single base resolution (80) already has inspired investigators within the chemical engineering field to design high-throughput microfluidics DNA-sequencing methods and instrumentation (96). The more complete mapping of differential energetic profiles for repair and replication pathways and their error-free and error-prone consequences remain to be determined.

Collectively, the above examples illustrate that additional energy profiling is required to more robustly bridge the gap between microscopic structural insights and macroscopic/cellular biological function/mechanisms. These energy profiles map the nature and relative strengths of the forces that regulate and control biological functions. Such characterizations create databases that can be mined to establish unique macroscopic signatures/correlations for biologically relevant properties (*e.g.*, mutagenicity *versus* genotoxicity) and, more generally, for energetically profiling successful and unsuccessful recognition, repair, and replication events. Failure in any of these events can result in mutations and, ultimately, cancers.

## Energy profiling of DNA triplet repeats: Rough DNA energy landscapes and dynamics, metastable substrate ensembles

**Figure 4:**
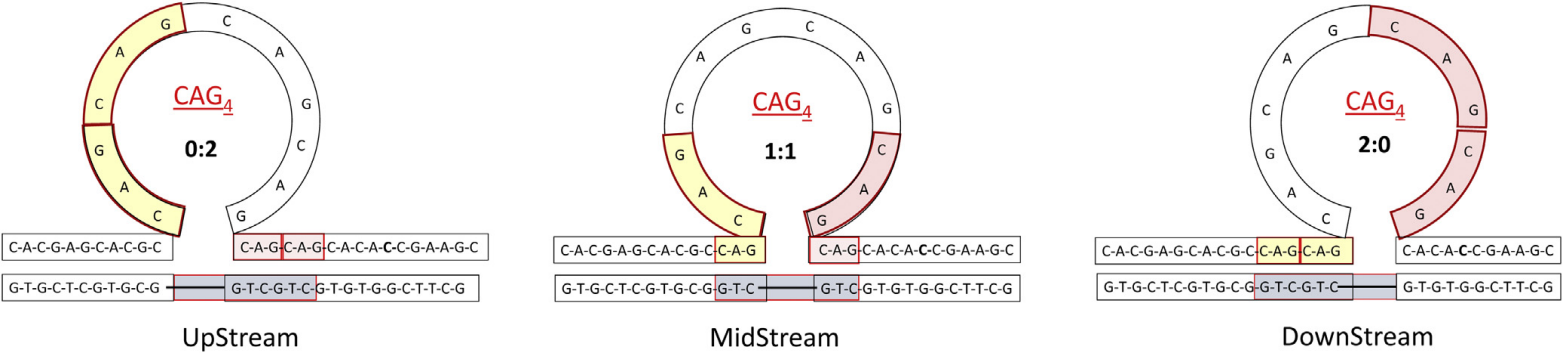
**Schematic of DNA triplet repeats being extruded out of a duplex as bulge loops that traverse along the helix to form a dynamic ensemble of metastable “rollamers” (****115****,****118****), whose properties depend on the size and sequence (****59****).**

Another example of connectivity between fundamental energy studies and biological structure and function is the determination of rough energy landscapes of metastably populated triplet repeat DNA systems. Such repeats are associated with families of neurodegenerative complex diseases, such as Huntington's disease, fragile X syndrome, and so forth (110, 111, 112, 113, 114). My laboratory has taken an interest in this topic, primarily because of the recruitment of Dr Jens Völker from Germany, by way of Professor Horst Klump's laboratory at the University of Cape Town in South Africa. Rather than focusing exclusively on the most stable states, Dr Völker developed calorimetric methods and designed DNA systems that would probe metastability/suboptimal states, a feature central to biological regulatory switches. His first interest focused on mapping the rough energy landscape of DNA triplet conformational gymnastics. To this end, he conducted calorimetric and spectroscopic studies on strategically designed and synthesized families of long oligonucleotides with different triplet repeat lengths and disease-inducing sequence repeats, flanked by duplex domains (59, 115, 116, 117), as illustrated in Figure 4.

These measurements revealed that triplet repeats are extruded out of the duplex as bulge loops and can traverse along the helix to form a dynamic ensemble of metastable “rollamers” (115, 118), whose properties depend on the size and sequence (59) (Fig. 4).

Beyond the intrinsic value of such studies, this family of unique DNA constructs provides an opportunity to map the interplay between kinetic and thermodynamic factors that collectively shape the rough energy landscapes of these molecules (119) as well as their recognition (120). Especially revealing is the dynamic nature of the DNA substrate, which differentially impacts the protein processing of such substrate ensembles. As such, beyond the thermodynamically most stable substrates, energy profiling also must consider kinetic factors that can trap metastable species of biological relevance.

By strategically inserting fluorescent probes and modifying mutagenic lesions at selective sites within the loops and the adjacent duplex domains, it was possible to probe and map intriguing crosstalk between DNA domains/regions (115, 121). These biologically consequential results required energy profiling and would not have been gleaned from structural studies alone. Significantly, it was revealed that rollamers with lesions within the loop were more stable than those with lesions in the duplex domains, thereby favoring the former family of “rollamers” within the ensemble.

Such *dynamic metastable substrate ensembles* (115, 118) present a challenge to the repair and transcription machinery that evolved to process fewer dynamic substrates (122, 123, 124). To this point, it has been demonstrated that CAG repeat bulge loops trap nonproductively a critical mismatch repair complex, MSH2–MSH3 (124, 125), which in turn stimulates components of the BER machinery, not normally associated with mismatch repair, causing these repeat domains to expand (126). Results from our laboratory provided a biophysical rationale for intriguing reports that activation of DNA repair induces expansion and pathogenicity of proximal DNA repeat domains, thereby yielding a basis for the rationale design of strategies for inhibiting triplet expansion associated with developmental disorders.

## Energy-based drug design

The concept of EBDD is an approach that further empowers and complements the paradigm of experimental and computational *structure-based drug design*. The missing structure/energy synergy was emphasized by Ray Salemme's quote in the cover story of Chemical & Engineering News (127), stating that “The initial expectation of structure-based design—that you were going to design molecules and they would work right out of the box—was unrealistic. We didn't understand the thermodynamics well enough.”

**Figure 5:**
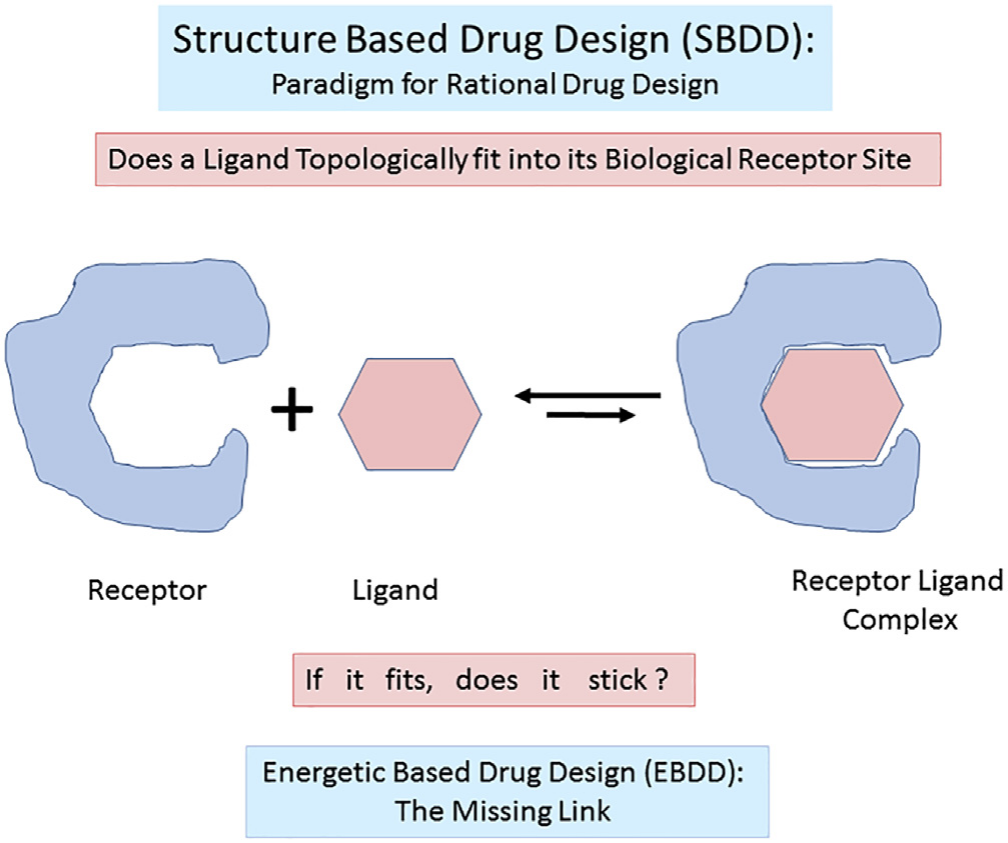
**Schematic of structure-based drug design complemented by energetic-based drug design.**

In other words, as cartooned in Figure 5, just because a drug fits topologically into its biological receptor, it does not mean that it functionally “sticks.” The latter assessment requires thermodynamic and kinetic binding profiles.

A number of laboratories have produced the binding energetics for a range of drugs that target protein and/or nucleic acid “receptors,” parsing the contributions of specific interactions by characterizing calorimetrically homologous series of ligands (128, 129, 130, 131, 132). Many of these studies further revealed that binding affinities alone do not provide the requisite insights for rational drug design needed to enhance lead optimization. Complete binding profiles (ΔG, ΔH, ΔS, ΔC_p_), preferably determined calorimetrically, are needed to elucidate the nature of the driving and/or inhibitory forces, including binding-induced and coupled changes in conformation and/or solvation (131, 132, 133). Changes in solvation have long been acknowledged as important features that strongly influence drug binding and target stability. However, not all thermodynamically perturbed solvent is sufficiently ordered so as to be detectable by structural techniques, ergo the need for parallel energy measurements that relate to alterations in solvation (134, 135), particularly heat capacity and volumetric assessments.

Fortunately, Armen Sarvazyan and Tigran Chalikian had joined my group from Armenia (in the former Union of Soviet Socialist Republics). They had pioneered the use of acoustic and densitometric techniques to define the solvation/desolvation impact on ligand affinity and target stability (136, 137, 138, 139, 140, 141) Their volumetric profiles provided unique insights into the impact of solvation changes as significant modulators of binding energetics (129, 142, 143). These data also provide an empirical basis for refining computational tools that predict and characterize binding energetics and dynamics with explicit incorporation of solvent contributions. Significantly, these collective energy characterizations correlate with the success or failure of a designed ligand to target its site and to express its biological activity (144, 145, 146).

In short, the enhanced power of parallel structure-based drug design and EBDD is now widely accepted by the scientific community. EBDD is now on an equal footing with the more traditional *structure-based drug design*, thereby enabling more effective drug discovery/screening protocols to help drive the development of new drugs. The importance of this reality is reflected in the large number of pharmaceutical and biotech companies that have incorporated isothermal titration and differential scanning calorimeters into their drug design and screening protocols (147, 148, 149, 150).

## Energy profiling of the genetic code: Implications for code evolution and molecular Darwinism

For many years, Klump, Völker, and I had been pondering how one could link fundamental DNA energetics with the shaping of the genetic code as an energy code (an *energy genome map*), ultimately correlating the resultant sequence-dependent genomic energy profiles with evolutionary trends. A very recent article from my laboratory addresses these interrelationships, revealing empirical correlations between codon energetics, genetic code evolution, and the concept of molecular Darwinism. In it, we demonstrate that one can employ a thermodynamic framework to rationalize the origins and evolution of the genetic code, and, more broadly, the genomic energy landscape (151). Specifically, we showed that differential DNA energy stability profiles correlate with differential biological outcomes. This observation is suggestive of the code functioning as an energy ladder comprised of linked thermodynamic cycles that evolved *via* sequential transitions and transversions, which selectively were driven by differential energy factors. In this context, we also propose that, in addition to the traditional Darwinian perspective of evolution, which is driven by natural selection due to phenotypic advantages, a form of “molecular Darwinism” also is at work, in which evolutionary pressures derive from energy optimization of a biophysical property. These recent results provide additional, more global examples of the unique insights revealed by fundamental energy profiling of complex biological processes.

To be specific, using nearest-neighbor DNA stability data, we translated the iconic chemical genetic code—the blueprint of life—into an energy code, revealing unique insights into two major universal enigmas in the life sciences. First, the energy mapping of the code explained how, out of an astronomical number of possible blueprints of life, the DNA genetic code evolved *via thermodynamic selection* into a nearly identical, singular blueprint for all living organisms.

Second, as alluded to above, this energy mapping of the genetic code expands the underpinnings of Darwin's natural selection/survival-of-the-fittest theory of evolution to include an additional component of molecular Darwinism. This concept of molecular Darwinism posits that some species characteristics evolutionarily persist across generations because their DNA energy codes are unusually stable, independent of whether or not the characteristics provide a survival advantage in a given environment, as proposed in the classic Darwinian evolutionary theory. Going forward, such energy mapping of the DNA chemical genome to create an *energy genome* will produce an enhanced roadmap for rationally designing DNA molecular targets that enable new diagnostic and therapeutic protocols.

## Concluding remarks

Mapping of energy profiles bridges the conceptual and practical gaps between biopolymer structures and biological functions. As elaborated in this *Reflections* article, such energy profiling for DNA is justified by unique energy-based insights that complement and expand elegant, structure-based understandings of DNA biological processes. Enhanced reporting of DNA energy profiling reflects the expanding recognition that such fundamental data are essential for foundational understanding of molecular recognition—and for the establishment of new biomedical insights and applications. DNA energy databases have enabled a revolution in quantitative molecular biology through the rational design of primers, probes, and arrays for diagnostic, therapeutic, and molecular-profiling protocols that have contributed to a myriad of biomedical assays that collectively impact human health.

I am very pleased to see how the foundational DNA energy data produced by my laboratory over nearly 5 decades have been, and continue to be, used for both fundamental understandings of molecular communication and for biomedically relevant, practical applications. Going forward, I am further encouraged by our DNA energy profiles/landscapes being used to empower the interpretations of complex new datasets produced by a myriad of emerging methodologies. The advent of increasingly sophisticated structural techniques (*e.g.*, single particle cryo-EM), some of which even provide time-resolved motion picture mapping of the dynamics associated with cellular processes (cryo-EM tomography), require enhanced computational methods to interpret the volumes of new primary structural/dynamic/functional data they yield. These computational methods are empowered when calibrated with experimental energetics such as those my laboratory is producing. Thus, aside from the intrinsic and application-based value of characterizing the intermolecular and intramolecular forces that modulate relative stabilities and molecular communications, calorimetry is having an increasing role in enabling the intensive computational methods required to interpret the rich information embedded within the primary data produced by these new methodologies (131).

Beyond enabling computational methods, we also now are focusing our energy profiling on complex, multicomponent biological machines—preferably machines in action, such as repair complexes or polymerase activities. These studies already include determining the comprehensive landscapes of complex dynamic systems, inclusive of kinetic effects, that modulate regulatory conformational switches (117). The methodologies required to accomplish such goals are at, or beyond, the limits of what one can do with existing instrumentation and analyses tools. Our pursuit of these goals will require the incorporation of methods in addition to calorimetry, as well as the development of different analysis methods to do justice to the complexities of such biological machines. Yet, the potential insights one can derive from comprehensive energy landscapes on complex biological machines more than justify the effort.

It is my hope that this *Reflections* article illustrates the personal and professional influences that shaped and motivated my five decades of contributions toward establishing DNA *Energy Profiling* as the co-equal sibling of *DNA Biological Structure and Biological Function*. As a “molecular linguist,” it has been and remains my fervent desire to establish DNA energy databases that characterize the words, sentences, paragraphs, and chapters that collectively constitute the language used in the book of molecular communication, thereby enabling the elucidation of the forces that dictate and control fundamental and complex biological processes. In the year 2071, I look forward to sharing with my colleagues the contributions from my second half-century of work!

## Conflict of interest

The author declares that he has no conflicts of interest with the contents of this article.
